# Association between household food insecurity and quality of life: a longitudinal study in Northeast Brazil, 2014–2019

**DOI:** 10.1186/s12889-026-28303-2

**Published:** 2026-07-03

**Authors:** Rônisson Thomas de Oliveira-Silva, Poliana de Araújo Palmeira, Rodrigo Pinheiro de Toledo Vianna, Ligia Rejane Siqueira Garcia, Klayton Galante Sousa

**Affiliations:** 1 Federal University of Paraíba (Health Sciences Center), João Pessoa, Paraíba Brazil; 2 Nutrition and Public Health Studies and Research Group (PENSO Group), Federal University of Campina Grande (Education and Health Center), Cuité, Paraíba Brazil; 3 Feral University of Paraiba (Graduate Program in Science of Nutrition - PPGCN/UFPB), João Pessoa, Paraíba Brazil; 4 Federal University of Rio Grande Do Norte (Faculty of Health Sciences of Trairi), Santa Cruz, Rio Grande Do Norte Brazil

**Keywords:** Food Insecurity, Quality of life, Health Vulnerability, Socioeconomic Factors, HRQOL

## Abstract

**Introduction:**

There are still some gaps in the knowledge about food insecurity (FI) as a determinant of quality of life (QoL); for example, it is not known whether there is a dose‒response relationship, whether these associations occur longitudinally, and how they behave in a population where the target audience is not composed of people affected by health problems.

**Methods:**

This work prospectively analyzed the association between QoL and FI in individuals living in a socioeconomically disadvantaged Brazilian municipality between 2014 and 2019. This was a longitudinal prospective cohort study involving 225 individuals from families residing in a municipality with high social vulnerability in the northeast semiarid region of Brazil. The present study considered QoL as the dependent variable and food insecurity (FI) as the main independent variable. Multivariate analyses were conducted using mixed-effects regression, separately for each QoL domain.

**Results:**

Baseline results showed mean QoL domain scores of 70.62 for Social Relationships, 69.42 for Physical Health, 64.77 for the Psychological domain, and 57.55 for the Environmental domain. Among these, only the Physical Health domain exhibited a statistically significant change at follow-up, with a mean reduction of 2.9 points. Multivariate analysis demonstrated an inverse association between FI and QoL scores in the Psychological and Environmental domains. Under conditions of moderate FI, scores declined by 4.728 points in the Psychological domain (*p* = 0.041) and 7.610 points in the Environmental domain (*p* = 0.000) over time. At the severe FI level, these reductions were more pronounced, reaching 9.465 points (*p* = 0.003) and 10.138 points (*p* = 0.000), respectively.

**Conclusion:**

The results presented in this cohort support the hypothesis that the phenomenon of FI was associated with poorer QoL outcomes over time.

**Supplementary Information:**

The online version contains supplementary material available at https://doi.org/10.1186/s12889-026-28303-2.

## Introduction

The World Health Organization defines Quality of Life (QoL) as an individual’s perception of their position in life within the context of the culture and value systems in which they live [1]. This definition has been widely disseminated and has become particularly relevant for its broader scope, as it transcends biomedical notions and aligns more closely with the concept of health [2, 3].

Currently, studies on QoL have increasingly examined the topic in relation to social determinants [4–6] or from a human rights–based perspective [7], suggesting that the guarantee or deprivation of such rights plays a crucial role in this outcome, as observed in the case of Food Insecurity (FI) [8–10].

FI is defined as the deprivation of, or uncertainty regarding, access to food in sufficient quantity and quality [11] and deserves special attention given its magnitude and global impact. In recent years, FI has intensified worldwide, with an estimated 828 million people experiencing its most severe level, namely hunger [12].

This hunger scenario negatively affects people’s QoL, as studies have shown that people with FI tend to have lower QoL [13–15]. However, there are still knowledge gaps about FI as a determinant of QoL, for example, regarding the existence of a dose‒response relationship and how this relationship unfolds longitudinally. Furthermore, most studies have been conducted with populations affected by health problems, such as chronic, infectious, and mental health conditions [16].

Based on this, the present study aimed to analyze the association between QoL prospectively, evaluated through the WHO quality of life scale abbreviated version (WHOQOL-BREF), and FI in individuals residing in a socioeconomically disadvantaged Brazilian municipality between 2014 and 2019.

## Method

### Study design and population

This is a longitudinal prospective cohort study whose sampling units are families residing in a municipality of high social vulnerability in the semiarid region of Northeast Brazil. The study was carried out in the municipality of Cuité, Paraíba, located 235.1 km from the state capital. The municipality has an estimated population of 20,334 people and a territorial area of 733,818 km^2^ [17].

The study sample was derived from a cohort of families initiated in 2011 (*n* = 358), with two follow-up stages: the first in 2014 (*n* = 326; 8.6% loss to follow-up) and the second in 2019 (*n* = 274; 15% loss to follow-up). For the baseline, a representative sample of the municipality's urban and rural populations was selected. The sample size was calculated using the stratified random sampling technique, dividing the municipality into urban and rural regions in proportion to their populations. A maximum sampling error of 5% was used, with a 95% confidence level and an expected prevalence of FI of 50%, the details of which are available in Palmeira et al. [18]. The cohort interviews were conducted by previously trained undergraduate students and were administered to the household's reference person, that is, the person identified by other family members as responsible for expenses, organization, or major decisions in the home. This practice is well established in large-scale household surveys in Brazil and was therefore adopted [19].

This study used data from the baseline (2014) and follow-up (2019) periods, which included the QoL assessment in the research instrument. The distribution of the sample across these two study periods is available in the supplementary material (Supplementary Table 1). Owing to the longitudinal analysis design, only families whose reference persons answered the questionnaire in both years were included in the final sample, resulting in 225 people. This sample size enabled the determination of associations between exposure (food insecurity) and outcome (quality of life) with 95% confidence.

Missing data derived from the application of the WHOQOL-BREF and *Escala Brasileira da Insegurança Alimentar* (EBIA—Brazilian Household Food Insecurity Measurement Scale) were disregarded from the analysis, and only those from the same 225 people who participated in all phases of the research were included. The MCAR test was performed to assess participant loss in the sample, and randomness (*p* = 0.287) was found in the loss of variables included in the study, demonstrating that there were no systematically missing differences.

### Dependent variable

#### Quality of life

The dependent variable in the present study was QoL, evaluated using the WHOQOL-BREF. This questionnaire was proposed by the WHO in the 1990s to assess the QoL of populations [20] and, since then, has been adopted by researchers worldwide as a reliable and valid measure to estimate the perception of QoL in different population groups [16, 21, 22]. The WHOQOL-BREF consists of 26 questions, 2 of which are general questions and the others address QoL from four domains: Physical Health (7 questions assessing the work capacity and willingness to perform activities of daily living, physical pain and dependence on medication or treatments), Psychological (6 questions about positive or negative feelings toward life, self-esteem and spirituality/religion/personal beliefs), Social relationships (3 questions about personal relationships, social support, and sexual activity), and Environment (8 questions addressing physical security, the home and physical environment, financial resources, social and health care, and access to information and transportation).

The items in the WHOQOL-BREF are all assessed with a Likert-type scale, with scores ranging from 1 to 5. QoL scores are estimated as the sum of the questions in each domain, ranging from 0 to 100, according to the methodology recommended by the WHO [20].

### Independent variables

#### Food insecurity

Given the importance and relevance of FI, studies have assessed it via scales, as highlighted by Marques et al. [23], who mapped 24 FI measurement instruments used in different parts of the world. In Brazil, the Brazilian Household Food Insecurity Measurement Scale (EBIA) was developed to estimate FI. It consists of 14 items and can provide a straightforward, low-cost, easy-to-apply measure of FI [24].

The EBIA is a scale adapted from the American instrument, the Household Food Security Survey Module, and has been validated for use in population-based studies in Brazil since 2003 [25]. The theoretical conception of this instrument considers food deprivation as a progressive phenomenon experienced at the household level and, in the most severe cases, at the individual level. The EBIA evaluates access to food in dimensions that include fear of suffering from food deprivation, reduced quality and/or quantity of food accessed by the family, and hunger [24].

The scale's 14 dichotomous (yes/no) items assess the family's lack of food over the last three months. Seven of these items are exclusive to families with at least one resident aged up to 18 years in the household. On the basis of the sum of affirmative answers, the classification of the FI of the families is made in terms of levels of severity, namely, (1) food security (FS)—when the participant answered negatively to the questions of the scale, which indicates that there was no fear or concern among family members of suffering food deprivation in the three months preceding the survey; (2) mild FI—when there is concern about experiencing food deprivation shortly; (3) moderate FI—when there is restriction in the quality of food; and (4) severe FI—when there was the experience of hunger among adults and/or children in the household.

For the analysis of FS and FI in a longitudinal approach, four categories of changes were created: "FS in both times" refers to families classified in FS both in 2014 and in 2019; "Changed to FS" characterizes the group of individuals who lived in households with FI in 2014 and overcame the situation by changing to FS in 2019; "Changed to FI" characterizes the group of individuals who lived in households in the FS situation in 2014 and experienced the worsening of the situation, moving to FI in 2019; and "FI in both times" characterizes the group of individuals who were in the FI situation both in 2014 and 2019.

Sociodemographic variables were adopted for sample description and adjustment, defined from the conceptual frame established by Kepple and Segall-Corrêa [24] and from the literature. Therefore, the following variables were included and categorized: age (adults; elderly age groups), sex (male; female), color/race (white; black/yellow/indigenous), area of residence (urban; rural), residence status (own and already paid; own and paying; assigned/rented/borrowed/other situations), per capita family income (Above ½ minimum wage; Between ¼ and ½ of the minimum wage; Up to ¼ of the minimum wage), education (High education; Average education; Low education) and availability of water at home (if it does have daily water).

### Data analysis

An exploratory analysis of the variables was performed using box plots, with estimates of measures of central tendency and sample dispersion for the four QoL domains in 2014 (baseline) and 2019 (follow-up).

Bivariate analyses were conducted to compare mean QoL scores by socioeconomic variables and FI. The inclusion of covariates for an adjusted model was guided by a conceptual model developed via Directed Acyclic Graphs (DAGs) and other studies in the literature [26–28]. Additionally, we considered the identified bivariate associations (*p* < 0.20) for each mean score of the QoL domains (Physical Health, Psychological, Social Relationships, and Environment).

The multivariate analysis consisted of a mixed-effects regression model, which was also performed separately for each QoL domain, including food insecurity, sociodemographic variables, and time as fixed effects. As a random effect, a random intercept was specified for each individual (id). In addition, robust standard errors were estimated to enhance the statistical inference.

In this model, the Physical Health domain was adjusted by the variables sex, age, education, and *per capita* income; the Psychological domain was adjusted by the variables sex, education, and per capita income; the Social Relationships was adjusted by sex, area of residence, and *per capita* income; and the Environment domain was adjusted by sex, area of residence, education and *per capita* income.

The regression coefficient (β) and the p-value were used to assess whether the FI categories were associated with QoL, with statistical significance defined as *p* < 0.05. Predicted QoL values by family FI levels were generated.

Collinearity tests were applied using the Variance Inflation Factor (VIF) to all regression models. VIF values ​​ranged from 1.02 to 1.63 (Physical Health), from 1.02 to 1.34 (Psychological), from 1.01 to 1.18 (Social Relationships), and from 1.02 to 1.35 (Environment), indicating the absence of significant multicollinearity among the covariates included in the model [29]. The statistical analysis was performed via the statistical program STATA (Stata Statistical Software) version 16.0.

### Ethical aspects

The research was submitted and approved by the Research Ethics Committee of the Alcides Carneiro University Hospital of the Federal University of Campina Grande/HUAC – UFCG, under number 30919314.6.0000.5182. All the respondents signed the Informed Consent Form for inclusion in the study.

## Results

### Socioeconomic characteristics and food insecurity

At baseline (2014), most participants were adults (73.78%), lived in urban areas (71.56%), were female (87.56%), black/yellow/indigenous (63.11%), had low or no education (65.77%), and had a mean age of 49 years (± 15.89). Only 6.31% of the participants had higher education, and 51.56% lived in families with per capita income above the ½ minimum wage (MW) (R$362.00). In the follow-up (2019), the percentage of adults was reduced to 61.78%, the average age increased to 54 years (± 16.00), and three families moved from rural to urban areas. The low schooling level was reduced to 62.16%, and the percentage of individuals who had completed higher education increased to 11.71%. There was also a small reduction in the percentage of families with per capita income above ½ MW (R$499.00). In addition, 67.65% of families who had a per capita income of up to ¼ of a MW in 2014 continued to receive up to ¼ of a minimum wage in 2019.

In 2014 (baseline), 63.11% of the studied population was classified as having FS, and 21.78%, 8% and 7.11% were classified as having mild, moderate, and severe FI, respectively. In 2019 (follow-up), 66.96% of the population had FS, and 18.75%, 9.82% and 4.46% had mild, moderate and severe FI, respectively. In addition, 50.67% and 20.89% of the families were classified into FS and FI at the two time points (2014 and 2019), respectively. With respect to the changes, 16% migrated from FI in 2014 to FS in 2019, and 12.44% migrated from FS to FI, worsening the situation.

### Quality of life

**Fig. 1:**
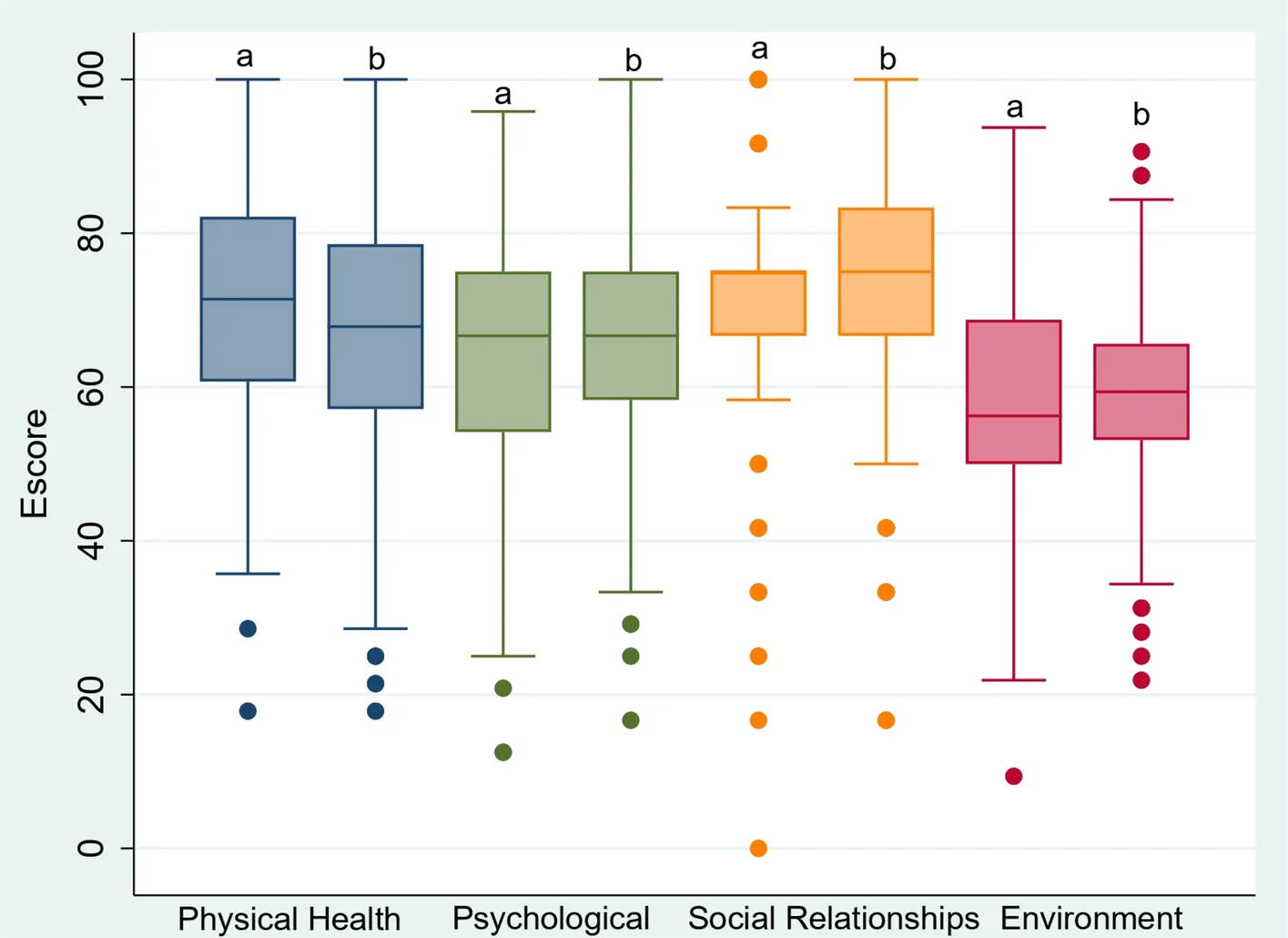
Medians and quartiles of the quality of life scores (0–100) for the Physical Health, Psychological, Social Relationships and Environment domains, in 2014^a^ (baseline) and 2019.^b^ (follow-up), Cuité, Paraíba, Brazil (*n* = 225)

Figure 1 below shows the medians and interquartile ranges for the four QoL scores among participants at the two study time points (2014 and 2019). It can be observed that the scores for three of these domains (Physical Health, Psychological Health, and Social Relationships) were between 60 and 80 in both years. The domain with the lowest score was Environment, which remained below 60, and the domain with the highest score was Social Relationships.

Furthermore, there was a significant reduction (*p* = 0.007) in the average score of the Physical Health domain between baseline and follow-up (−2.9), and there was an increase, without significance, in the Psychological (+ 1.47), Social Relationships (+ 0.97), and Environment (+ 0.79) domains.

The study results also demonstrated that the mean QoL domain scores over the two years studied were unevenly distributed according to participants’ sociodemographic characteristics. At baseline (Table 1), women presented significantly lower mean scores across all QoL domains (Physical Health = 68.69; Psychological = 63.72; Social Relationships = 69.54; Environment = 56.26). Similarly, individuals with lower educational attainment showed significantly lower scores in the Physical Health (67.88) and Psychological (63.18) domains.

**Table 1:** Sociodemographic characteristics and food insecurity according to quality of life scores (0–100) for the Physical Health, Psychological, Social Relationships and Environment domains in 2014, Cuité, Paraíba, Brazil (*n* = 225)

*Characteristics*	Physical Health		Psychological		Social Relationships		Environment	
	Mean ± SD	p	Mean ± SD	p	Mean ± SD	p	Mean ± SD	p
Age group								
18–59	71.10 ± 14.09	**0.004**^**a**^	65.03 ± 15.10	0.666^**a**^	70.68 ± 16.86	0.935^**a**^	56.98 ± 13.24	0.257^**a**^
> 60	64.70 ± 16.27		64.05 ± 14.63		70.48 ± 15.18		59.16 ± 10.80	
Sex								
Male	74.61 ± 13.44	**0.049**^**a**^	72.17 ± 9.75	**0.004**^**a**^	78.27 ± 14.40	**0.008**^**a**^	66.62 ± 11.38	**0.000**^**a**^
Female	68.69 ± 15.01		63.72 ± 15.28		69.54 ± 16.41		56.26 ± 12.33	
Color/race								
White	67.34 ± 14.91	**0.108**^**a**^	65.11 ± 14.86	0.799^**a**^	70.78 ± 14.68	0.914^**a**^	58.32 ± 11.16	0.489^**a**^
Black/yellow/indigenous	70.64 ± 14.85		64.58 ± 15.06		70.53 ± 17.38		57.10 ± 13.48	
Education								
Low education	67.88 ± 14.35	**0.034**^**b**^	63.18 ± 15.24	**0.066**^**b**^	70.20 ± 14.96	0.342^**b**^	56.74 ± 12.22	0.334^**b**^
Medium education	71.19 ± 16.13		67.54 ± 14.94		70.16 ± 19.93		58.82 ± 14.30	
High education	77.55 ± 9.33		69.94 ± 9.69		76.78 ± 9.90		60.93 ± 9.70	
*Per capita* income (minimum wage)								
Up to ¼	69.46 ± 13.97	0.342^**b**^	64.73 ± 14.70	**0.062**^**b**^	70.04 ± 15.54	0.222^**b**^	54.48 ± 11.33	**0.006**^**b**^
Between ¼—½	66.42 ± 13.90		60 ± 14.02		67.08 ± 17.18		55.46 ± 12.45	
Above the ½	70.44 ± 15.77		66.45 ± 15.18		72.19 ± 16.55		60.10 ± 13.04	
Area of residence								
Urban	69.78 ± 15.29	0.568^**a**^	64.64 ± 15.25	0.837^**a**^	70.60 ± 16.58	0.966^**a**^	57.82 ± 12.68	0.617^**a**^
Rural	68.52 ± 14.04		65.10 ± 14.30		70.70 ± 16.05		56.88 ± 12.69	
Food Insecurity								
FS	71.42 ± 14.69	**0.039**^**b**^	67.13 ± 13.76	**0.000**^**b**^	71.94 ± 16.14	0.254^**b**^	60.32 ± 11.83	**0.000**^**b**^
Mild FI	67.56 ± 14.65		62.92 ± 14.98		70.23 ± 16.40		55.22 ± 12.30	
Moderate FI	63.09 ± 15.78		63.42 ± 15.75		66.20 ± 17.72		52.08 ± 12.26	
Severe FI	64.50 ± 14.31		51.04 ± 17.17		65.10 ± 16.72		46.28 ± 12.76	

MW (minimum wage); Low education (people without schooling or with incomplete primary education); Medium education (people with complete primary education or incomplete secondary education or complete secondary education); High education (people with complete/incomplete technical course or complete/incomplete higher education)

^a^Student's t test

^b^analysis of variance (ANOVA)

Furthermore, a relevant finding was observed regarding FI: participants experiencing any of the three levels of FI had lower scores across all QoL domains compared with those classified as FS. Similarly, individuals with per capita income below half the minimum wage had significantly lower scores in the Psychological and Environmental domains. Additional results are presented below.

When analyzing the average QoL domain scores by sociodemographic variables during follow-up (Table 2), similarities were observed with the baseline findings, with the worst average scores for women across all domains (Physical Health = 65.53; Psychological = 65.65; Social Relationships = 71.10; Environment = 57.85). Those with low education levels continued to show significantly lower values ​​in the Physical Health (mean = 64.72) and Psychological (mean = 64.91) domains, but in this second phase of the research also presented lower average scores in the Environment domain (mean = 57.47).

**Table 2:** Sociodemographic characteristics and food insecurity according to quality of life scores (0–100) for the Physical Health, Psychological, Social Relationships and Environment domains in the year 2019, Cuité, Paraíba, Brazil (*n* = 225)

*Characteristics*	Physical Health		Psychological		Social Relationships		Environment	
	Mean ± SD	p	Mean ± SD	p	Mean ± SD	p	Mean ± SD	p
Age group								
18–59	69.70 ± 16.08	**0.000**^**a**^	67.11 ± 12.62	0.235^**a**^	71.88 ± 14.51	0.709^**a**^	58.47 ± 11.93	0.839^**a**^
> 60	61.37 ± 17.76		64.82 ± 16.08		71.12 ± 15.26		58.13 ± 12.30	
Sex								
Male	73.46 ± 14.20	**0.022**^**a**^	70.38 ± 12.12	**0.095**^**a**^	75 ± 14.16	**0.192**^**a**^	65.06 ± 11.18	**0.001**^**a**^
Female	65.53 ± 17.38		65.65 ± 14.24		71.10 ± 14.83		57.39 ± 11.89	
Color/race								
White	67.59 ± 15.40	0.474^**a**^	67.01 ± 14.59	0.527^**a**^	72.48 ± 13.57	0.487^**a**^	59.18 ± 12.37	0.425^**a**^
Black/yellow/indigenous	65.89 ± 18.17		65.78 ± 13.76		71.06 ± 15.46		57.85 ± 11.87	
Education								
Low education	64.72 ± 18.40	**0.048**^**b**^	64.91 ± 14.64	**0.024**^**b**^	71.61 ± 15.71	0.559^**b**^	57.47 ± 12.33	**0.046**^**b**^
Medium education	67.73 ± 15.28		67.52 ± 12.50		70.25 ± 13.61		58.62 ± 11.82	
High education	73.48 ± 13.06		72.75 ± 11.06		74.03 ± 12.98		63.82 ± 9.31	
*Per capita* income								
Up to ¼ of the MW	69.08 ± 17.49	0.269^**b**^	64.72 ± 14.13	**0.021**^**b**^	71.26 ± 16.38	0.275^**b**^	55.98 ± 12.29	**0.013**^**b**^
Between ¼—½ of the MW	63.82 ± 19.05		62.96 ± 15.91		69.29 ± 16.80		56.25 ± 12.52	
Above ½ MW	66.67 ± 15.98		68.86 ± 12.43		73.13 ± 12.44		60.79 ± 11.14	
Area of residence								
Urban	66.24 ± 17.20	0.691^**a**^	65.82 ± 14.47	0.471^**a**^	70.07 ± 14.87	**0.011**^**a**^	57.69 ± 12.46	**0.185**^**a**^
Rural	67.27 ± 17.27		67.34 ± 12.93		75.68 ± 13.80		60.09 ± 10.75	
Food Insecurity								
FS	67 ± 16.51	0.789^**b**^	68.13 ± 13.04	**0.002**^**b**^	72.22 ± 13.26	0.254^**b**^	60.22 ± 11.38	**0.000**^**b**^
Mild FI	66.32 ± 19.75		65.27 ± 15.79		72.22 ± 16.22		57.81 ± 11.75	
Moderate FI	63.14 ± 18.91		56.43 ± 15.19		65.53 ± 22.16		48.86 ± 14.86	
Severe FI	68.21 ± 13.72		65.83 ± 7.80		72.5 ± 8.82		53.43 ± 4.02	

MW (minimum wage); Low education (people without schooling or with incomplete primary education); Medium education (people with complete primary education or incomplete secondary education or complete secondary education); High education (people with complete/incomplete technical course or complete/incomplete higher education)

^a^Student's t test

^b^analysis of variance (ANOVA)

In addition, both those with some level of financial independence and those with per capita income less than half the minimum wage continued to show significantly lower scores in the Psychological and Environment domains. Another relevant result from this table was that those living in rural areas had better average scores in the Social Relationships (mean = 75.68) and Environment (mean = 60.09) domains.

### Associations between FI and QoL

Table 3 presents the medium scores of each QoL domain according to changes in FS or FI status between baseline (2014) and follow-up (2019). Of the 225 participants, the results showed that 114 (50.67%) remained in a state of FS at both time points assessed. In addition, 36 individuals (16%) overcame the FI condition and moved to FS. Conversely, 28 participants (12.44%) who were in FS at baseline moved to FI, indicating a worsening of their condition. Finally, 47 individuals (20.89%) remained in FI at both time points analyzed.

**Table 3:** Comparison of medium quality of life scores according to change in categories of food insecurity between 2014 and 2019, Cuité, Paraíba, Brazil (*n* = 225)

Quality of life domains	FS both moments		*p*	Changed to FS		*p*	Changed to FI		*p*	FI both moments		*p*
	2014	2019		2014	2019		2014	2019		2014	2019	
Physical Health	72.36 ± 14.85	66.94 ± 16.38	**0.009**	67.75 ± 15.30	67.16 ± 17.17	0.877^a^	67.60 ± 13.64	67.98 ± 17.77	0.928^a^	64.66 ± 14.38	64.13 ± 19.02	0.878^a^
Psychological	67.50 ± 13.87	69.18 ± 12.74	0.341^a^	62.15 ± 16.31	64.81 ± 13.60	0.454^a^	65.62 ± 13.44	63.24 ± 17.89	0.575^a^	59.66 ± 16.04	61.96 ± 13.69	0.455^a^
Social Relationships	73.09 ± 14.58	73.31 ± 12.74	0.903^a^	70.13 ± 15.85	68.75 ± 14.41	0.698^a^	67.26 ± 21.02	71.72 ± 12.89	0.342^a^	67.02 ± 17.37	69.50 ± 19.75	0.519^a^
Environment	61.21 ± 10.99	60.96 ± 10.98	0.865^a^	54.94 ± 12.03	57.89 ± 12.44	0.309^a^	56.69 ± 14.42	56.58 ± 12.33	0.975^a^	51.19 ± 13.10	53.39 ± 12.63	0.410^a^

^a^Paried Student’s t test

Comparison of the means indicated that the only domain with a statistically significant difference was Physical Health (*p* = 0.009) for those individuals who were in FI at both time points. Furthermore, it was found that the "FI both moments" group presented the lowest average scores in the four QoL domains.

In the multivariate mixed-effects analysis, FI showed a negative association with different domains of QoL, revealing that the greater the severity of FI, the greater the impact on average scores. In situations of moderate FI, scores in the Psychological and Environmental domains decrease by −4,728 and −7,610 points, respectively. In the case of severe FI, this decrease is −9,465 points (Psychological) and −10,138 points (Environmental) (Table 4).

**Table 4:** Mixed effects multivariate regression model results for scores of the quality of life domain and food insecurity, 2014–2019, Cuité, Paraíba, Brazil

Exposure variables	**Physical Health**		**Psychological**		**Social Relationships**		**Environment**	
	β	p	β	p	β	p	β	p
Mild FI	−1.451	0.447	−2.029	0.239	0.201	0.919	−1.952	0.184
Moderate FI	−2.151	0.448	−4.728	**0.041**	−5.887	0.060	−7.610	**0.000**
Severe FI	−2.866	0.356	−9.465	**0.003**	−3.129	0.319	−10.138	**0.000**

Adjusted for:

Physical Health domain outcome: sex, age, education, *per capita* income

Psychological domain outcome: sex, education, *per capita* income

Social Relationships domain outcome: sex, area of residence, *per capita* income

Environment outcome: sex, area of residence, education*, per capita* income

Although the coefficients for the Physical Health and Social Relationships domains also showed a negative trend with increasing FI severity, these associations did not reach statistical significance (*p* < 0.05). These results suggest that higher levels of FI are especially related to worse psychological and environmental conditions in the studied population.

**Fig. 2:**
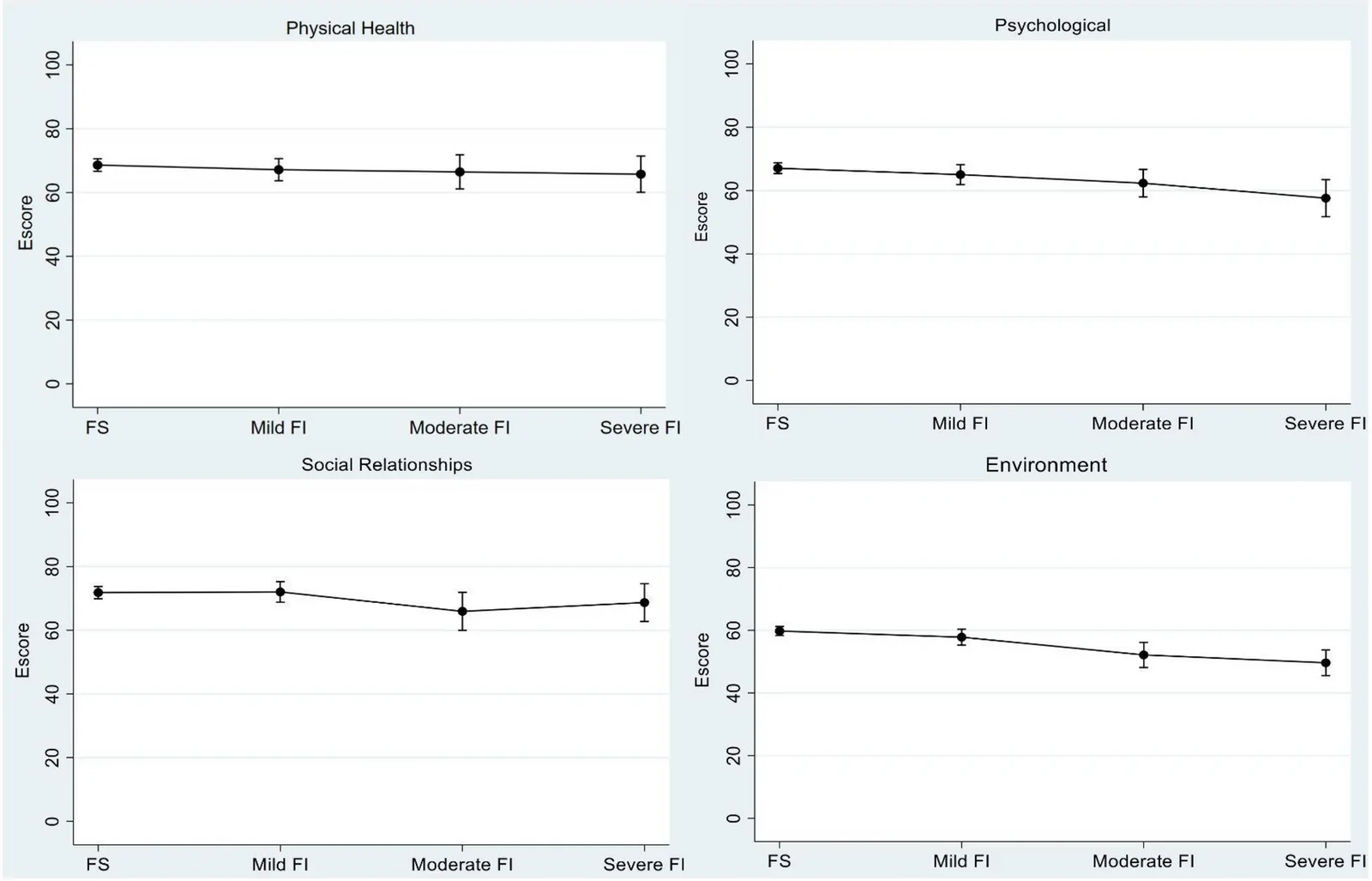
Predicted QoL domain scores according to FI situation and severity

Figure 2 presents the predicted QoL score values according to the FI situation and severity estimated from the results in Table 4. In the Psychological domain, the predicted mean scores are similar for the FS, mild and moderate FI categories and decrease significantly in the severe FI situation. In the case of the Environment domain, the predicted mean scores were lower in the moderate and severe FI categories than in the FS and mild FI categories. In the Physical Health and Social Relationships domains, this effect of FI in reducing the score was not observed.

## Discussion

This study presented longitudinal data on the QoL of a population residing in a context of high social vulnerability in Northeast Brazil. The results demonstrated that the studied sample presented average QoL scores in the four domains that were worse than those observed for a population of adults and elderly people in southeastern Australia [30] and similar to the findings of a survey of adults and elderly people conducted in a capital city in the Northeast region of Brazil [31].

Furthermore, the findings showed that the domain with the worst score was the Environment, which was also observed in the study by Purba et al. [32] in a general population in Indonesia, and the domain with the best evaluation was Social Relationships, corroborating the studies by Singh et al. [33] in rural communities in Bangladesh and by Singh et al [34] in a vulnerable elderly population in India.

This domain of Social Relationships assesses the individual's perception of social support. This support makes individuals feel cared for and loved and can improve their ability to cope with stressful events [35]. Interlenghi and Salles-Costa [36] observed that effective support helps alleviate stress caused by “worry or uncertainty about having enough to feed a family”.

Importantly, the mean scores for the QoL domains remained stagnant over time, showing no statistically significant differences, except for the Physical Health domain, which showed a reduction. Hammoudeh [37] also observed this absence of changes in a 4-year interval with Palestinians.

Furthermore, they revealed that this distribution of average scores across the four domains occurs unevenly, since the scores were significantly worse for women in all domains and in both years of the research, as well as for people with low levels of education, lower per capita income, and who resided in households with food insecurity.

These differences in QoL outcomes have been reported in the literature, including gender differences. Studies conducted in low- and middle-income countries have indicated that among the reasons for this phenomenon are lower social status, lower wages, barriers to accessing healthcare, double work shifts, and greater responsibility in the home environment [14, 38].

Given that the majority of the studied sample consists of women and that gender inequality in QoL levels was demonstrated, it is important to highlight the need for public policies to address this situation. Santos et al [39] conducted research in the same municipality studied and identified that there were 26 Food and Nutritional Security Initiatives in 2014, and that, among these, 10 had their activities interrupted in 2019 (five inactive and five extinct), revealing a federal dismantling in the implementation of these policies, weakening the fight for gender equality and contributing to the findings of the present study.

Additionally, the main finding of this study was the estimate that FI, at its moderate and severe levels, was associated with lower mean QoL scores in the Psychological and Environmental domains. Furthermore, a gradient relationship was observed, with greater FI severity corresponding to a greater reduction in scores across these domains. These findings support an important longitudinal association that, until now, had not been documented in the literature.

Regarding the association between FI and QoL domains, the study results were similar to those reported by Batista et al. [31], revealing an association between FI and worse perception in the assessment of the Psychological and Environmental domains.

In contrast to the present study's results, some studies have reported adverse effects of FI on Physical Health. However, some of these studies analyzed people who were ill, as in the case of the study by Ghahramanloo et al. [40] with type 2 diabetes patients or the study by Palermo et al. [41] with people living with HIV, which may potentiate the association between this domain and FI.

As a central finding of the present study, FI, especially severe FI, was associated with lower scores in the Psychological and Environmental domains over the years.

The explanatory mechanisms for this association between FI and the Psychological domain stem from the anxiety, chronic stress, and pressure that this condition of limited access to food causes in people's mental health [42–45].

Similarly, Gyasi, Obeng, and Yeboah [46] reported that FI at moderate and severe levels significantly increased the psychological distress score, providing evidence to support the hypothesis that starvation increases psychological distress.

The study by Cole and Tembo [47] examined relationship between FI and mental health in a rural population in Zambia. The authors concluded that the subjects lived with worries and anxieties because, in the dry season, several families that could not harvest and stockpile food worried about ensuring basic goods and about delays in the next harvest. This study contributes to the understanding that there may be a fundamental difference in psychological factors between urban and rural populations.

Notably, mental health is affected by various levels of FI, ranging from fear of having no food to eating to experiencing hunger. The EBIA used in this study to assess FI in households raises structural questions that address concerns about difficulties accessing food, corroborating the observed relationship between FI and lower scores in the Psychological domain.

In the Environment domain, both severe and moderate FI were associated with lower scores. The questions in the WHOQOL-BREF address the physical environment, financial resources, leisure opportunities, access to health services, transportation, and related areas. These factors are directly related to family income, to the social development of a country, and the planned public policies. If poverty rates increase, for example, society is likely to face situations of social vulnerability, triggering health problems and worsening QoL. According to Pérez-Escamilla [48], poverty is the primary determinant of FI, just as FI is one of the main determinants of poverty.

Thus, a possible explanatory mechanism for this association is that lower individual income and higher levels of population poverty contribute to a context of food insecurity which, especially in its moderate and severe forms, negatively impacts the perception of environmental dominance. This scenario also imposes an unequal struggle for survival on the entire family [49] and weakens citizenship [50].

Another important aspect is that although Brazil made progress between 2000 and 2015 in reducing wage inequality and overcoming poverty, in 2016 the country faced an unstable political context resulting from the presidential impeachment, marking a series of setbacks that led to an economic and political crisis [51]. This scenario caused a decline in purchasing power, increased unemployment, and higher product inflation, directly deteriorating the food and nutritional security situation of the Brazilian population and consequently affecting the aspects of QoL related to the Environment domain.

From this, there is a need, emphasized in Brazil by the field of public health, to assess subjective health and explore the impact of these sociodemographic and economic conditions on the associated outcomes [37, 52, 53]. QoL measures have been proven to help analyze, for example, the experiences of vulnerable populations [54], minority populations [55], and populations in highly stressful situations [37], and it is argued that this study fulfilled that objective.

One limitation of this study is selection bias due to loss to follow-up, although this was determined to be random based on appropriate statistical tests. Furthermore, the time interval between the two assessment points limits the depth of the analysis, since families can undergo several changes over 5 years. Another limitation is related to the use of the "household's reference person" who, although indicated by other family members as the one who best understands the dynamics of the home, is recognized as a potential source of information bias for subjective variables, such as QoL, since the scores obtained predominantly reflect the individual perception of these participants.

Finally, an important factor concerns the presence of acute illnesses among the interviewed individuals. In the present study, it was not possible to investigate and evaluate the influence of acute illnesses on QoL, which constitutes a limitation of the study but, at the same time, also contributes to clarifying a substantial gap observed in the literature on the subject: the absence of studies that analyze the longitudinal associations between FI and QoL in general populations.

## Conclusion

This study achieves its objective of prospectively evaluating the association between FI and QoL. The results support the hypothesis that FI was associated with lower average scores across QoL domains over time. It was observed that, especially at its most severe level, FI was associated with a reduction in average scores in the Psychological and Environmental domains of QoL, demonstrating a relevant longitudinal association.

The results provide important evidence for the field of public health by evaluating QoL in a general sample, not limited to people with a disease, with longitudinal follow-up, and by contributing to reflection on the importance of a governmental agenda focused on overcoming social inequalities, food and nutritional security, and improving the QoL of vulnerable communities.

## Supplementary Information

Supplementary Material 1.

## Data Availability

The datasets used and/or analysed during the current study are available from the corresponding author on reasonable request.

## Abbreviations

FI: Food Insecurity
FS: Food Security
QoL: Quality of Life
WHO: World Health Organization
UN: United Nations
FAO: Food and Agriculture Organization
EBIA: Escala Brasileira da Insegurança Alimentar
