# Supplementary material for: Association between household food insecurity and quality of life: a longitudinal study in Northeast Brazil, 2014–2019
Source: BMC Public Health. 2026 Jul 3;26:2669. doi: 10.1186/s12889-026-28303-2 (PMC13602778; doi:10.1186/s12889-026-28303-2)
Supplement: Supplementary file 1 [file 12889_2026_28303_MOESM1_ESM.docx]

**Supplementary table**

**Supplementary Table 1.** Sociodemographic characteristics, Food Insecurity and Quality of Life in the years 2014 (n=326) and 2019 (n=274), Cuité, Paraíba, Brazil.

| ***Characteristics*** | **Baseline (2014)** | | **Follow-up (2019)** | | ***p*** |
| --- | --- | --- | --- | --- | --- |
|  | n (%) | | n (%) | |  |
| **Age group** |  | |  | |  |
| 18-59 | 244 (74.85) | | 174 (64.44) | | < 0.001^a*^ |
| >60 | 82 (25.15) | | 96 (35.56) | |  |
| **Sex** |  |  | | | |
| Male | 48 (14.72) | | 44 (16.30) | | 0.144^a^ |
| Female | 278 (85.28) | | 226 (83.70) | |  |
| **Color/race** |  | |  |  | |
| White | 117 (36.34) | | 95 (35.32) | | 0.617^a^ |
| Black/yellow/indigenous | 205 (63.66) | | 174 (64.68) | |  |
| **Education** |  | |  |  | |
| Low education | 204 (64.15) | | 168 (63.16) | | 0,002^b*^ |
| Medium education | 29 (29.25) | | 66 (24.81) | |  |
| High education | 21 (6.60) | | 32 (12.03) | |  |
| ***Per capita* income** |  | |  |  | |
| Up to ¼ of the MW | 107 (32.82) | | 70 (25.74) | | 0.077^b^ |
| Between ¼ - ½ of the MW | 62 (19.02) | | 67 (24.63) | |  |
| Above ½ MW | 157 (48.16) | | 135 (49.63) | |  |
| **Area of residence** |  | |  |  | |
| Urban | 224 (68.71) | | 200 (72.99) | | 0.205^a^ |
| Rural | 102 (31.29) | | 74 (27.01) | |  |
| **Food Insecurity** |  | |  |  | |
| FS | 202 (61.96) | | 178 (65.20) | | 0.477^b^ |
| Mild FI | 73 (22.39) | | 51 (18.68) | |  |
| Moderate FI | 27 (8.28) | | 28 (10.26) | |  |
| Severe FI | 24 (7.36) | | 16 (5.86) | |  |
|  |  | |  | |  |
| **Quality of Life** | **Mean ± SD** | | **Mean ± SD** | | ***p*** |
| Physical Health | 69.54 **±** 14.95 | | 67.20 ± 16.93 | | 0.049^c*^ |
| Psychological | 65.41 **±** 15.08 | | 66.46 ± 13.79 | | 0.090^c^ |
| Social Relationship | 71.69 ± 16.01 | | 71.67 ± 15.23 | | 0.599^c^ |
| Environment | 58.10 ± 12.28 | | 58.18 ± 12.25 | | 0.44^c^ |

^a^McNemar Test; ^b^Stuart-Maxwell Test; ^c^T test. *The results were considered statistically significant when	p<0.05
